# The assessment of P-wave dispersion and myocardial repolarization parameters in patients with chronic kidney disease

**DOI:** 10.1080/0886022X.2017.1419962

**Published:** 2017-12-29

**Authors:** Korhan Kollu, Lutfullah Altintepe, Cevdet Duran, Mustafa Topal, Samil Ecirli

**Affiliations:** a The Department of Internal Medicine, Konya Health Application and Research Center, University of Health Sciences, Konya, Turkey;; b The Division of Nephrology and Internal Medicine, Konya Health Application and Research Center, University of Health Sciences, Konya, Turkey;; c The Deparment of Internal Medicine, The Division of Endocrinology and Metabolism, Usak University, The School of Medicine, Usak, Turkey;; d The Division of Internal Medicine, Konya Health Application and Research Center, University of Health Sciences, Konya, Turkey

**Keywords:** P-wave dispersion**;** QTc dispersion**;** Tp-e dispersion**;** Tp-e/QT ratio**;** chronic kidney disease**;** arrhytmias

## Abstract

**Objective:** The risks of sudden death and cardiac arrhythmia are increased in patients with chronic kidney disease (CKD). Here, we aimed to evaluate the indicators of arrhythmias, such as p-wave dispersion (P-WD), QTc dispersion, Tp-e and Tp-e/QT ratio in patients with CKD stages 3–5 on no renal replacement therapy (RRT).

**Material and methods:** One-hundred and thirty three patients with CKD stages 3–5 and 32 healthy controls were enrolled into the study. No patients received RRT. QTc dispersion, P-WD and Tp-e interval were measured using electrocardiogram and Tp-e/QT ratio was also calculated.

**Results:** Mean age rates were found similar in patients and controls (60.8 ± 14.2 and 61 ± 12.9 y, *p* = .937, respectively). Compared patients with controls, P-WD (45.85 ± 12.42 vs. 21.17 ± 6.6 msec, *p* < .001), QTc-min (366.99 ± 42.31 vs. 387.15 ± 20.5 msec, *p* < .001), QTc dispersion (71.13 ± 27.95 vs. 41.25 ± 14.55 msec, *p* < .001), Tp-e maximum (81.04 ± 10.34 vs. 75.49 ± 10.9 msec, *p* < .001), Tp-e minimum (62.25 ± 7.58 vs. 54.8 ± 6.72 msec, *p* < .001) and Tp-e/QTc ratio (0.19 ± 0.02 vs. 0.18 ± 0.01, *p* = .001) were found to be different. QTc-max and Tp-e interval were found to be similar in both groups.

**Conclusion:** P-WD and QTc dispersion, Tp-e interval and Tp-e/QTc ratio were found to be increased in with CKD stages 3–5 on no RRT.

## Introduction

Chronic kidney disease (CKD) is among important health problems across the world, and it is known that cardiovascular disease (CVD) is a major cause of morbidity and mortality in patients with CKD [1,2]. Left ventricular hypertrophy, myocardial fibrosis, coronary artery disease, changes seen in the concentrations of serum electrolytes such as calcium and potassium and increased frequency of such disorders as diabetes mellitus (DM), hypertension and hyperlipidemia may be contributing factors to the increase in CVD in these patients [3,4]. Many different noninvasive electrocardiograhy (ECG)-based methods have been recommended to detect the risk of arrhytmia in patients with CKD and general population. As one of these methods, P-wave dispersion (P-WD) has come to the fore in the prediction of atrial fibrillation (AF) [5,6] and cardiovascular mortality [7] in patients with CKD. P-WD is defined as the difference between the maximum (P_max_) and minimum (P_min_) durations of P-wave and can be measured easily with ECG. In addition, it has been reported that P-WD increased in patients with CKD exposed to renal replacement therapy (RRT), compared to the controls [8] and that the increase continued after hemodialysis, compared to predialysis values [9–11]. Intracardiac calcification including the atria is known as a common finding in patients with CKD and may lead to impaired cardiac function [12,13]. Hypertensive cardiomyopathy, an important effect on left atrial structure and function, is mostly seen in hemodialysis patients [13]. Structural cardiac changes, which are distinctive for patients with chronical renal disease, might have an effect on P-wave indices.

Also, P-WD has been reported to be associated with the decrement of renal functions [14]. On the other hand, there are studies with controversial findings, reporting that hemodialysis has no effect on P-WD [15] or on-line hemodiafiltration has various benefical effects on P-WD [16]. Likewise, another important predictor of life-threatening ventricular arrhythmias [17], the dispersion of QT interval is a marker of alterations in ventricular repolarization and described as the difference between the maximum (QT_max_) and minimum QT (QT_min_) intervals on ECG. It is reported that the durations of QT and corrected QT (QTc) dispersion increase in hemodialysis and peritoneal dialysis patients [18–20] and hemodialysis has negative effects on QTc dispersion [18,19].

Tp-e interval, an another arrhythmogenesis predictor, is measured as the distance from the peak of the T-wave to the end point and Tp-e/QT ratios are reported to be associated with increased ventriculary arrhythmogenesis [21–23]. In addition to the high prevalence of coronary artery atheroma [24], diabetic dialysis patients were shown to have a reduced coronary flow reserve (CFR) in the absence of coronary vessel stenoses in predialysis patients [25]. CFR determines the ability to increase blood flow to the myocardium during increased demand and there is preliminary evidence that the same reduction of CFR is also seen in dialysis patients without diabetes [26]. Hemodialysis patients characteristically also exhibit left ventricular (LV) hypertrophy, reduced peripheral arterial compliance, impaired microcirculation [27] and ineffective vasoregulation (in response to hemodialysis with ultrafiltration). All of these factors also predispose to demand ischemia. Apart from conventional risk factors of sudden cardiac death studied in the general population, patients with chronical renal disease have distinct underlying pathologies predisposing them to these events and possibly have different relative impacts. Among these are myocardial hypertrophy, left ventricular diastolic dysfunction, myocardial fibrosis, microvessel disease, dialysis-induced myocardial injury and stunning, disorders of mineral metabolism and secondary hyperparathyroidism. Additionally, the dialysis procedure itself, mainly via rapid electrolyte shifts, may increase the arrhythmic risk, not only during the session but also in the long run [28,29].

Tp-e interval and the ratio of Tp-e/QT still remain unclear entities in CKD patients without RRT. In this study, we aimed to evaluate P-WD, QTc dispersion, Tp-e and Tp-e/QT ratio in patients with CKD at stages 3–5 not on RRT in order to assess atrial and ventriculary repolarisation.

## Material and methods

This prospective study was conducted in the division of nephrology in Konya Health Application and Research Center, University of Health Sciences between September 2014 and March 2015. Informed consent was obtained from all participants. The study protocol was approved by the ethics committee of Meram Medical Faculty of Necmettin Erbakan University.

One hundred and thirty-three patients with CKD (79 men) and 32 healthy controls (20 men) were enrolled into the study. Prior to and during the study period, no patients with CKD underwent RRT. Those taking antiarrythmic, antiparasitic drugs or antibiotics that may affect cardiac repolarization, having left or right bundle-branch block on ECG, left ventricular ejection fraction ratio of lower than 60% or those with any known active infection, benign or malignant hematologic disorders, any solid tissue cancers or exposed to any surgical intervention within the past six months were excluded from the study.

Blood samples were drawn after an overnight fasting to measure blood urea, creatinine, sodium, potassium, uric acid, magnesium and albumine, centrifuged and stored in deep freeze at −70 °C until being analyzed. Complete blood count was measured by Sysmex XE-2100 (Sysmex Corp, Kobe, Japan) with flourescence flow cytometry or electrical impedance method. Blood urea, creatinine, sodium, potassium, uric acid, magnesium and albumin levels were measured with spectroschopic method by Abbot C16000 autoanalyser (Abbot Laboratories, Abbot Park, IL). Estimated glomerular filtration rate (eGFR) levels (mL/min/1.73 m^2^) were calculated using the formula of Modification of Diet and Renal Disease [30].

Twelve-lead ECGs (Nihon Kohden Corporation, model ECG-1350 K, Tokyo, Japan) at the speed of 25 mm/s were obtained from all patients, while resting in the supine position. Heart rates were calculated by averaging three consecutive RR intervals on DII derivation. All measurements on ECG were made manually with calipers by two observers (KK and LA). The rates of P_max_ and P_min_ durations were measured and P-WD was calculated through the difference between P_max_ and P_min_ durations [5]. The QT intervals were calculated for each derivation by measuring from the beginning of the QRS complex to the end of the T wave off-set, as defined by the return of the terminal T wave to isoelectric TP baseline and QTc was corrected for heart rate using the Bazzett's formula [31]. The QTc dispersion was calculated via the difference between maximum (QTc-max) and minimum (QTc-min) QTc intervals. The Tp-e interval was measured as the averages of all leads from the peak of T wave to the end [32]. The Tp-e/QT ratio was also calculated by dividing Tp-e by QT.

### Statistical analyses

The statistical analyses of the data were carried out with SPSS v15 statistical software package. The normality of the data was analyzed with the Shapiro-Wilk test. The descriptive statistics for variables with normal distribution of continuous data [mean ± standard deviation (SD)] and with no normally distributed variables [median (minimum:maximum)] were indicated. The independent-samples *t* test for normally distributed continuous data and the Mann-Whitney *U* test for non-normally distributed continuous data were used in the comparison of two independent groups. The chi-squared test was used to assess the differences between categorical variables. Each variable that showed a correlation with a *p* ≤ .1 was entered into a binary logistic regression model as a potential predictor. This approach led to a formulas expressing the laboratory and demografic parameters that were predictive of differences between patients and controls groups. Parameters that were shown to be predictors in a formulas were used to construct the formulas of binary logistic regression analysis. The relationship between the parameters was determined using the Pearson’s and Spearman coefficient of correlation. The multivariate linear regression analysis was used to identify the independent predictors of prolonged Tp-e interval, increased Tp-e/QT ratio, increased P-WD and independent variables differing significantly in the bivariate analyses (*p* < .1). *p* value < .05 was considered to be significant. Statistically significant values are indicated in bold in the Tables 1–3.

**Table 1.** Demographic characteristics of study population.

	Patients (*n* = 133)	Controls (*n* = 32)	*p* value^a^
Age (y)	60.8 ± 14.2	61.0 ± 12.9	.937
Gender (M/F)	79/54	20/12	.842
Diabetes Mellitus (*n*, %)	52 (39.1%)	None	
Hypertension (*n*, %)	92 (69.2%)	None	
Coronary artery disease (*n*, %)	17 (12.8%)	None	
eGFR (ml/min)	25.04 ± 12.1	84.28 ± 15.7	**<.001**
Creatinine (mg/dL)	2.93 ± 1.36	0.88 ± 0.14	**<.001**
Sodium (mEq/L)	137.63 ± 3.2	136.37 ± 3.23	.054
Potassium (mEq/L)	4.99 ± 0.67	4.23 ± 0.34	**<.001**
Calcium (mg/dL)	8.77 ± 0.75	9 ± 0.62	.076
Phosphorus (mg/dl)	3.83 ± 0.9	3.28 ± 0.6	**<.001**
Albumin (g/dL)	3.9 ± 0.42	3.92 ± 0.47	.805
Uric acid (mg/dL)	7.16 ± 1.63	5.17 ± 1.33	**<.001**
Hemoglobline (g/dl)	12.37 ± 2.03	13.32 ± 2.05	**.022**

a Significant results are given in bold.

eGRF: estimated glomerular filtration rate.

**Table 2.** Electrocardiography parameters of study population.

	Patients (*n* = 133)	Controls (*n* = 32)	*p* value^a^
P_max_ (msec)	106.88 ± 14.08	94.28 ± 7.20	**<.001**
P_min_ (msec)	61.02 ± 11.15	67.10 ± 4.35	**.003**
P-WD (msec)	45.85 ± 12.42	27.17 ± 6.6	**<.001**
QTc-max (msec)	438.12 ± 49.06	428.40 ± 23.4	.104
QTc-min (msec)	366.99 ± 42.31	387.15 ± 20.5	**<.001**
QTc (msec)	411.1 ± 26.2	415.8 ± 23.0	.319
QTc disp (msec)	71.13 ± 27.95	41.25 ± 14.55	**<.001**
Tp-e max (msec)	81.04 ± 10.34	75.49 ± 10.9	**.012**
Tp-e min (msec)	62.25 ± 7.58	54.8 ± 6.72	**<.001**
Tp-e interval (msec)	71.7 ± 8.4	66.5 ± 7.4	**.001**
Tp-e disp (msec)	18.79 ± 7.56	20.69 ± 9.87	.314
Tp-e/QT ratio	0.19 ± 0.02	0.18 ± 0.01	**.001**

P_max_: maximum p wave duration; P_min_: minimum p-wave duration; P-WD: p-wave dispersion; QTc-max: maximum corrected QT duration; QTc-min: minimum corrected QT duration; QTc disp: QTc dispersion; Tp-e max: maximum Tp-e duration; Tp-e min: minimum Tp-e duration.

a Significant results are given in bold.

**Table 3.** Bivariate correlation and multivariate linear regression analyses between increased Tp-e/QT ratio (average of all leads) and study parameters.

	Bivariate correlation		Multivariate linear regression	
Parameters	*r*	*p* value	β	*p* value^a^
Uric acid (mg/dL)	−0.164	.060	−0.156	.712
Calcium (mg/dL)	0.224**	.009	0.894	.414
Phosphorus (mg/dl)	−0.229**	.008	0.139	.875
Potassium (mEq/L)	−0.263**	.002	−2.711	**.013**
Hemoglobline (g/dl)	0.320**	<.001	0.116	.817
i-PTH	−0.335**	<.001	−0.015	**.002**
eGFR (ml/min)	0.290**	.001	0.029	.704
Age (years)	−0.110	.210	–	–
Male	−0.056	.520	–	–
Diabetes Mellitus	0.028	.751	–	–
Hypertension	0.058	.508	–	–
Coronary artery disease	0.088	.315	–	–
Albumin (g/dL)	0.109	.210	–	–
Sodium (mEq/L)	0.046	.597	–	–
Magnesium (mg/dL)	−0.112	.217	–	–
CRP (mg/dl)	−0.012	.893	–	–
Ferrite (mg/dl)	−0.075	.390	–	–

CRP: C-reactive protein; eFGR: estimated glomerular filtration rate; i-PTH: intact parathyroid test

a Significant results are given in bold.

## Results

A total of 165 participants, 133 patients with CKD (79 men) and 32 healthy controls (20 men), constituted our study population. Prior to and during the study period, no patients with CKD underwent RRT. The mean ages of cases were similar in the patients and controls. Summarized in Table 1, the following parameters such as hemoglobin (*p* = .022), serum creatinine (*p* < .001), potassium (*p* < .001) and uric acid (*p* < .001) were found to be higher in the patient group, compared to the controls.

Leading to CKD, the following underlying etiological factors were detected: hypertensive nephropathy in 54 patients (32.7%), diabetic nephropaty in 45 (27.3%), chronic glomerulonephritis in 11 (6.7%), obstructive uropathy in 10 (6.1%), policystic kidney disease in seven (4.2%), chronic tubulointerstitial nephritis and amiloidosis in two patients (1.2%) and another two (1.2%) and unknown etiology in two (1.2%) patients, respectively.

The ECG analyses revealed that 126 (94.7%) patients had normal sinus rythm, four (3%) patients had atrial extrasystole and three (2.3%) had ventricular extrasystole.

The results of ECG parameters are summarized in Table 2. P_max_ values were determined to be higher in the patient group, compared to the controls, while P_min_ values were seen to be higher in the controls. P-WD value was found to be higher in the patients than the controls (45.85 ± 12.42 vs. 27.17 ± 6.6 msec, *p* < .001). Although similar QTc-max and higher QTc-min values were found in the controls than the patients, the values of QTc dispersion were found to be higher in the patients, compared to the controls (71.13 ± 27.95 vs. 41.25 ± 14.55 msec, *p* < .001). Tp-e interval, Tp-e maximum and Tp-e minimum were found to be higher in the patients group. Tp-e/QT ratio was also found to be higher in the patients, compared to the controls (0.19 ± 0.02 vs. 0.18 ± 0.01, *p* < .001).

Correlation analyses demonstrated that P-WD dispersion was positively correlated with uric acid (*r* = 0.289, *p* < .001), sodium (*r* = 0.194, *p* = .012), potassium (*r* = 0.256, *p* = .001), QTc dispersion (*r* = 0.172, *p* = .027) and Tp-e/QT (*p* = .195, *r* = 0.012) and negatively correlated with eGFR (*r* = −0.318, *p* < .001). It was also shown that Tp-e was positively correlated with calcium (*r* = 0.211, *p* = .007), hemoglobin (*r* = 0.171, *p* = .028), QTc dispersion (*r* = 0.303, *p* < .001) and Tp-e/QT ratio (*r* = 0.643, *p* < .001) and that Tp-e/QT ratio was positively correlated with hemoglobine (*r* = 0.224, *p* = .004), P-WD (*r* = 0.195, *p* = .012) and QTc dispersion (*r* = 0.223, *p* = .004). QTc dispersion was found to be positively correlated with creatinine (*r* = 0.342, *p* < .001), uric acid (*r* = 0.159, *p* = .043), P-WD (*r* = 0.172, *p* = .027), Tp-e (*r* = 0.303, *p* < .001) and Tp-e/QT ratio (*r* = 0.223, *p* = .004), while negatively correlated with eGFR (*r* = −0.353, *p* = .019).

In the comparison of the patients and controls, 93 (69.9%) cases in the patient group were seen to have prolonged P-WD duration, while those in the controls had no prolonged P-WD duration (>40 msec; *p* < .001). It was also seen that 108 (81.2%) cases in the patients and eight (25%) cases among the controls had prolonged QTc dispersion duration (>50 msec; *p* < .001), while no cases in the patients and only one (3.1%) case in the controls had prolonged Tp-e interval duration (>110 msec; *p* = .194). Also, two (1.5%) cases in the patients were observed to have increased Tp-e/QT ratio, while no case in the controls had increased Tp-e/QT ratio (>0.25; *p* = .649).

Based on the presence of DM, our patients were re-divided into two subgroups as those with and without DM and such parameters as P-WD, QTc dispersion, Tp-e duration and Tp-e/QT ratio were found to be similar in both groups.

As a result of multivariate linear regression analysis, the higher potassium (β = −2.711, *p* = .013) and i-PTH (β = −0.015, *p* = .002) were the independent predictors of an increased Tp-e/QT ratio (Table 3). In addition, there was a significant correlation between the Tp-e/QT ratio in the averages of all of the leads, and calcium (*r* = 0.224, *p* = .009) phosporus (*r* = −0.229, *p* < .008), potassium (*r* = −0.263, *p* = .002), hemoglobine (*r* = 0.320, *p* < .001), i-PTH (*r* = −0.335, *p* < .001) and eGFR (*r* = 0.290, *p* = .001).

Likewise, in multivariate linear regression analysis, the higher potassium (β= −2.633, *p* = .012) and i-PTH (β = −0.017, *p* = .001) were also demonstrated to be the independent predictors of a prolonged Tp-e interval. In addition, a significant correlation was present between the Tp-e interval in the averages of all of the leads and albumine (*r* = 0.225, *p* = .009), calcium (*r* = 0.290, *p* = .001), phosporus (*r* = −0.175, *p* = .044), potassium (*r* = −0.257, *p* = .003), hemoglobine (r = 0.296, *p* = .001), i-PTH (r = -0.373, *p* < .001) and eGFR (r = 0.260, *p* = .003). However, no parameters predicted an increased P-WD in multivariate linear regression analysis and also no parameters were correlated with P-WD (Table 4).

**Table 4.** Bivariate correlation and multivariate linear regression analyses between increased P-WD (average of all leads) and study parameters.

	Bivariate correlation		Multivariate linear regression	
Parameters	*r*	*p* value	β	*p* value
Uric acid (mg/dL)	0.046	.600	–	–
Calcium (mg/dL)	−0.124	.154	–	–
Phosphorus (mg/dl)	−0.096	.273	–	–
Potassium (mEq/L)	−0.002	.983	–	–
Hemoglobine (g/dl)	−0.085	.328	–	–
i-PTH	−0.038	.668	–	–
eGFR (ml/min)	0.096	.273	–	–
Age (years)	−0.134	.124	–	–
Male	0.007	.933	–	–
Diabetes Mellitus	<0.001	1	–	–
Hypertension	0.001	.988	–	–
Coronary artery disease	0.15	.189	–	–
Albumin (g/dL)	−0.047	.589	–	–
Sodium (mEq/L)	0.132	.130	–	–
Magnesium (mg/dL)	−0.109	.228	–	–
CRP (mg/dl)	−0.067	.455	–	–
Ferrite (mg/dl)	0.062	.478	–	–

CRP: C-reactive protein; eFGR: estimated glomerular filtration rate; i-PTH: intact parathyroid test

All of the 12 candidate parameters that distinguished between two outcomes with a *p* < .10 were entered into a logistic regression model as candidate predictors (Table 5).

**Table 5.** Results of binary logistic regression models differentiating between patients and controls groups.

Compared Outcomes	Models	% Correct	r^2^	Variables	*p* value	Exp β (OR)	95% CI of Exp β
Patients?control groups	Y = 23.45 − 1.288 × uric acid −3.478 × potassium	96.9	0.842	Uric acid	.003	0.266	0.110–0.645
				Potassium	.003	0.031	0.003–0.299

CI: confident interval; OR: odds ratio.

## Discussion

In the present study, the values of P-WD, QTc dispersion and Tp-e/QT ratio were found to increase in predialysis patients with stages 3–5 CKD on no RRT.

Increased P_max_ and P-WD duration are known to increase the risk of atrial fibrilo flutter [5]. In a study performed by Dilaveris et al. [5], a cutoff value is recommended as 110 msec (88% of sensitivity and 83% of specifity) for P_max_ and as 40 msec (75% of sensitivity and 85% of specifity) for P-WD in order to distinguish the healthy controls from the patients. In another study by Solak et al. [8], when compared to the controls, P-wave index was reported to become increased in CKD patients on hemodialysis and in those on continuous ambulatory peritoneal dialysis (CAPD), but the increase was significant in those on hemodialysis, while insignificant in those on CAPD. In the same study, the prevalance rates of intraatrial block in CKD patients on hemodialysis and CAPD, and controls were reported to be 61, 55 and 32%, (*p* = .001), respectively. In another study, Huang et al. [33] reported that increased P-WD and P_max_ duration are associated with faster decline in renal functions, increased requirement for dialysis or even with increased risk of death in stages 3 or 4 and end-stage CKD patients. Unver et al. reported that ultra filtrate volume greater than 1 L increased P-WD values during hemodialysis [34]. As consistent with the findings in previous studies, the durations of P_max_, P_min_ and P-WD were found to increase in CKD patients with stages 3–5 on no RRT in our study. As a limitation in our study, we performed no long-term ambulatory cardiac rhytym analysis and so no comparison of P_max_, P_min_ and P-WD with the risk of arrhythmia was carried out. However, considering the results of previous studies, we consider that the increased rates of P_max_, P_min_ and P-WD may be seen in CKD patients and speculate that the increase may trigger or be responsible for the risk of arrhythmia in these patients.

The durations of QT and QTc and QTc dispersion were found to increase in the patients on dialysis, compared to the controls [18–20]. Morris et al. [19] reported that the duration of QTc max and QT dispersion increased in the patients with CKD on hemodialysis and that the rise continued after the hemodialysis. In various studies, different factors are emphasized to have effects on QT dispersion in the patients on hemodialysis. Afshinnia et al. [35] reported that low magnesium dialysate used during hemodialysis had no effects on QT dispersion in hemodynamically stable hemodialysis patients. In another study, Tong et al. [20] detected that an increase was present in the duration of QT and QTc dispersion after hemodialysis and that as the reason for the increase, a decrease in sodium and potassium, an increase in calcium and ultra filtrate volume affected QT and QTc dispersion in these patients during hemodialysis. In the study by Jaroszynski et al. [18], the changes of serum calcium, phosphorus, potassium and extracellular volume during hemodialysis were reported to affect QTc dispersion and to promote ventricular arrythmogenesis. On the other hand, it was also shown that renal transplantation had a beneficial effect on QTc duration and maximum QTc interval was shorter in the patients with renal transplation, compared to the patients on hemodialysis [36]. Our study population was composed of the patients with CKD stages 3–5 on no RRT. However, QT min and QTc dispersion were found to be increased in our patients with CKD, compared to the controls. As a limitation of our study, we found no result for the significance of prolonged QTc, because we didnot investigate cardiac rhythm analysis. When considering the results of previous studies, it may be speculated that prolonged QTc dispersion has an effect on the cardiac mortality and morbidity rates in the patients with CKD.

In recent studies performed in different groups of patients with such diseases as left ventricular hyperthropy, hyperthropic cardiomyopathy and implantable cardioverter defibrillator, Tp-e/QT ratio has been suggested to be more beneficial in the evaluation of ventricular repolarization and arrythmogenesis, compared to Tp-e interval and QTc [32,37,38]. Despite the similar findings of Tp-e interval in the patients with CKD stages 3–5 on no RRT, higher Tp-e/QT ratio was found in our patients with CKD stages 3–5 on no RRT, compared to the controls. To the best our knowledge, there is no study evaluating Tp-e and Tp-e/QT ratio in the patients with CKD stages 3–5 on no RRT. In a single study published by Tun et al. [39], Tp-e was reported to be increased in the patients with ESRD, compared to the controls. In another study reporting no changes in QTc interval and QT dispersion, it was reported that Tp-e interval and Tp-e/QT ratio increased after hemodialysis and also speculated that hemodialysis induced ventricular arrythmias [40]. Because the number of studies is too limited, we consider that no speculation can be made on Tp-e interval and Tp-e/QT ratio in the patients with CKD and that these entities should be supported with novel studies with larger sample size.

As another finding of our study, no difference was observed between diabetic and non-diabetic populations in terms of the duration of P-WD, QTc and Tp-e interval and also Tp-e/QT ratio. It is a known fact that DM increases the risk of ventricular arryhmogenesis [41]. In a study published by Tokatli et al. [42], the values of Tp-e, Tp-e/QT ratio were reported to increase in patients with type 2 DM.

However, our study has some liminations. Firstly, we evaluated no relationship between arrhythmias and the predictors of arrthythmias, such as PWD, QTc, Tp-e and Tp-e/QT; therefore, we acquired no information about the effects of such parameters on the patients with CKD. Secondly, because our patients were on no RRT, no effects of RRT on these parameters were investigated in our study. Thirdly, due to technical reasons, blood PH and ionized calcium could not be measured in both groups, while magnesium, C-reactive protein (CRP) and intact parathyroid hormone (i-PTH) levels were not measured in the controls. Finally, based on literature, whether various factors, especially drugs used in pre-dialysis patients, have effects on Tpe still remains unclear.

In conclusion, our study showed that as the predictors of arrhythmias and sudden cardiac deaths, the durations of P-WD and QTc dispersion and also Tp-e/QT ratio were increased in the patients with CKD on no RRT. The screening of CKD patients on the basis of PWD, QTc dispersion and Tp-e/QT ratio, which can be measured with ECG easily, could help detect the patients with high risk profiles and prevent worse outcomes like cardiac arrhythmias and sudden deaths.
